# Investigations on Agglomeration and Haemocompatibility of Vitamin E TPGS Surface Modified Berberine Chloride Nanoparticles

**DOI:** 10.1155/2014/951942

**Published:** 2014-08-04

**Authors:** Parameswara Rao Vuddanda, Vijayakumar Mahalingam Rajamanickam, Madhu Yaspal, Sanjay Singh

**Affiliations:** ^1^Department of Pharmaceutics, Indian Institute of Technology (Banaras Hindu University), Varanasi, Uttar Pradesh 221005, India; ^2^Department of Anatomy, Institute of Medical Sciences, Banaras Hindu University, Varanasi, Uttar Pradesh 221005, India

## Abstract

The objective of the present study is to investigate the influence of surface modification on systemic stability of NPs. Vitamin E TPGS (1% w/v) was used for surface modification of berberine chloride nanoparticles. Naked and surface modified NPs were incubated in different SBFs (pH 6.8 and 7.4) with or without bile salts and human plasma. NPs were observed for particle agglomeration and morphology by particle size analyzer and TEM, respectively. The haemocompatibility studies were conducted on developed NPs to evaluate their safety profile. The surface modified NPs were stable compared to naked NPs in different SBFs due to the steric stabilization property of vitamin E TPGS. Particle agglomeration was not seen when NPs were incubated in SBF (pH 6.8) with bile salts. No agglomeration was observed in NPs after their incubation in plasma but particle size of the naked NPs increased due to adhesion of plasma proteins. The TEM images confirmed the particle size results. DSC and FT-IR studies confirmed the coexistence of TPGS in surface modified NPs. The permissible haemolysis, LDH release, and platelet aggregation revealed that NPs were compatible for systemic administration. Thus, the study illustrated that the surface modification is helpful in the maintenance of stability of NPs in systemic conditions.

## 1. Introduction

Recent days, pharmaceutical research field has paid more interest to utilization of nanotechnology for the development of potential dosage forms [1–5]. Nanotechnology based formulations have demonstrated great unique advantages in terms of their affinity, efficacy, and targetability over conventional dosage forms [6–11]. In this view, formulation scientists have also developed various theories and strategies for improvement of productivity, quality, safety, and elegance of nanosize pharmaceutical products [12–16]. Several reports suggest that preservation of particle integrity of nanosize products is necessary until it reaches a specific site of action in body. The particle integrity influences the stability of nanoproducts and plays an important role in achieving the expected benefit from the nanoparticulate carriers. The tendency of particle aggregation, sedimentation, and physicochemical incompatibility of starting materials may lead to instability of nanoformulations. The Brownian motion and surface charge of nanoparticles (NPs) are majorly responsible for maintenance of stability at either storage or systemic circulation. Employing stabilizer (surfactants) in NPs preparation avoids particle aggregation due to electrostatic repulsive mechanism and surface tension property. Lyophilisation has also been used for removal of water from nanoformulation in order to maintain stability at storage conditions [17–19]. Therefore, the use of stabilizers (surfactants) and lyophilization of NPs are widely practiced techniques for preservation of particle integrity and stability during preparation as well as storage conditions [20]. In spite of the vast evidence on the importance of different surfactants in maintaining the stability of NPs during preparation and storage conditions which have been widely documented, limited information is available about its effect in maintenance of systemic stability of NPs. In fact, systemic stability of NPs is an important issue while designing formulation for site specific delivery or targeting. Otherwise, the aggregation of particle will lead to changes in its size and thus ultimately fail to deliver the benefits of nanosized formulations. Therefore, the maintenance of nanoparticle stability in the both storage and systemic conditions is still essential and should be addressed by the researchers.

Iijima and Kamiya reported that surface modification is advisable approach for preservation of stability of NPs at certain pH conditions [21]. Surface modification phenomenon is the latest approach for improving retention time and longevity of NPs in systemic circulation. It is commonly done by using hydrophilic property substances. Currently, polyethylene glycols (PEG) and its derivatives are greatly incorporated in several formulations for surface modification to provide longevity to NPs in the systemic circulation [22–25]. However, these can also be useful in providing stability to the NPs in the GIT and systemic circulation is not well established. Based on this rational, the present research work is focussed on investigating the influence of surface modification on agglomeration behaviour of nanoparticles at different simulated biological fluids (pH 6.8 and 7.4) with or without bile salts and human plasma. Berberine chloride loaded polymeric nanoparticles were taken as model nanoparticles. Berberine chloride (BBR) is a traditional plant alkaloid and mentioned in Indian Ayurveda and Chinese Medicine for its traditional antimicrobial and antiprotozoal properties. Present research on BBR has also revealed various pharmacological properties and medicinal uses which are beneficial in the treatment of diabetes, cancer, depression, hypertension, and hypercholesterolemia [26–30]. Despite these multimedicinal uses, BBR has poor bioavailability (less than 5%) as its uptake is inhibited by P-gp efflux system. It has been reported that delivery of drugs which are P-gp substrates in nanoencapsulation form could be helpful for improvement of bioavailability through circumvention of P-gp efflux system [31, 32]. Therefore, development of nanoparticulate formulation of BBR would be beneficial in increasing its bioavailability and efficacy by improving its absorption. D-Alpha-tocopheryl polyethylene glycol 1000 succinate (vitamin E TPGS), a PEG derivative, is selected as surface modifying agent. It is water soluble derivative of natural vitamin E as nonionic nature amphilic copolymer existed with lipophilic alkyl tail and hydrophilic polar head. The highly hydrophilic nature provides increased cellular uptake, prolonged blood circulation, and enhanced bioavailability. Specifically, it enhances drug permeability and absorption of drugs by inhibiting the P-glycoprotein. It has been also reported as a stabilizer and surface modifier in drug delivery for different types of cancer treatment [33–36]. NPs will be subjected to different pH's in GIT and blood during its absorption and circulation, respectively. Two of the simulated biological fluids (SBFs) have been selected for this study as they mimic the intestine and blood environment of the body. The sodium taurocholate (20 mM) was selected as it mimics the bile salt secretion in GIT. In addition, hemocompatibility properties such as haemolysis, LDH release, and platelet aggregation were also estimated in human blood as a primary tool for prediction of safety profile of developed surface modified nanocarriers.

This overall study was also conducted relatively with naked nanoparticles for better understanding of surface modification efficiency in systemic condition. Further, this study will provide additional information about vitamin E TPGS effect on blood components which otherwise had not been documented during the time of conceptualization of this study. This information may be useful in establishing its safety profile and effective use in pharmaceutical applications.

## 2. Materials and Methods

### 2.1. Materials

Berberine chloride was gift sample received from Inga Pharmaceuticals, Mumbai, India. Polycaprolactone (PCL), molecular weight of ~45,000 g/mol, was procured from Sigma Aldrich, USA. Sodium taurocholate (STC) and Pluronic F-68 (F-68) were kind gifts of Newzeland Pharma (Newzeland) and BASF (India), respectively. Water used in all the experiments was ultrapure water obtained from a Millipore-DirectQ UV ultrapure water system, Millipore, France. Other chemicals of analytical grade were purchased locally and used as received.

### 2.2. HPLC Analytical Method

A reverse-phase, high-performance liquid chromatography (HPLC) analytical method was used for quantification of BBR [37]. The HPLC system consisted of a 515 HPLC binary pump (Waters, USA), a Rheodyne 7725i manual injector (Waters, USA), Photodiode Array Detector (Waters, USA), and the operating software Empower Node 2054. Drug quantification was carried out using a C_18_ reverse-phase (250 × 4.6 mm, 5 mm) ODS2 column (Waters Corp., Milford, MA, USA). The mixture of acetonitrile and 0.02 M phosphoric acid in the ratio of 45 : 55 v/v was used as mobile phase. The mobile phase was filtered through a 0.45 *μ*m millipore filter and degassed prior to use. The flow rate was 1.0 mL/min. Detection was performed at a wavelength of 346 nm (*λ* max) and the column was maintained at a constant temperature (25 ± 1°C). Standard curves were constructed in the range of 1–20 *μ*g/mL (triplicate) and validated for linearity, precision, accuracy, limit of quantification (LOQ), and limit of detection (LOD).

### 2.3. Preparation of Naked and Surface Modified Nanoparticles

Naked nanoparticles were prepared following nanoprecipitation method described by Singh and Muthu [38]. In brief, the PCL was dissolved in acetone (organic phase) at 40°C and added into ethanolic solution of BBR. The organic phase was dropped into the aqueous phase containing 50 mM of F-68 (stabilizer) at the rate of 6 mL/min using syringe equipped with needle under magnetic stirring speed (800 rpm) at 25°C. Then, the acetone was evaporated under reduced pressure at 40°C using a rota evaporator (IKA, Germany). The obtained nanosuspension was centrifuged at 15,000 rpm for 30 min and then nanoparticles pellet was separated.

The surface modification of the BBR NPs was carried out by a method described in Kulkarni and Feng [39]. The BBR NPs were mixed and incubated in 1% w/v aqueous solution of vitamin E TPGS for overnight at room temperature. The surface modified nanoparticles were separated by centrifugation at 15,000 rpm for 30 min at −4°C (Remi, India). The obtained naked and surface modified nanoparticle pellets were lyophilized using lyophilizer (Lyophilizer, Decibel, India) for 36 h at −40°C. Both types of lyophilized nanoparticles were stored in cool condition (8°C) until further use.

### 2.4. Incubation of Nanoparticles in Simulated Biological Fluids and Human Plasma

Both types of NPs (equivalent 10 mg of drug) were primarily dispersed in 1 mL of water separately to make nanoparticle dispersion and incubated in 9 mL of simulated biological fluids of pH 7.4 and 6.8 with or without 20 mM of sodium taurocholate and human plasma for 30 minutes at 37°C.

### 2.5. Particle Size, Polydispersity Index, and Zeta Potential

The particle size (PS), polydispersity index (PDI), and zeta potential (ZP) were estimated by Delsa Nano C (Beckmann Coulter, USA). The PS and PDI were measured by dynamic light scattering technique at 25°C. Samples were scattered at an angle of 165°. Data were fitted by the method of inverse “Laplace transformation” and CONTIN. Polydispersity index indicates the distribution of particle size of nanoparticles. The ZP of nanoparticles was measured from the electrophoretic mobility under electric field using Helmholtz-Smoluchowski equation.

### 2.6. Entrapment Efficiency

The entrapment efficiency (EE) was estimated with method described by Bisht et al. [40]. The nanosuspension around 500 *μ*L was placed in the upper chamber of Nanosep centrifuge tubes containing ultrafilter with molecular weight cut-off 100 KD (Pall Life Sciences, India). Nanosep was centrifuged at 5000 rpm for 30 minutes using cooling centrifuge (Remi, C-24, India) at −4°C. The filtrate was collected from lower chamber and unentrapped BBR was estimated by HPLC method. The EE was calculated by the following equation:

(1) $$\begin{array}{c} \text{EE}\left(\%\right)=\left[\frac{A_{t}-A_{\text{un}}}{A_{t}}\right]\times 100, \end{array}$$

where *A*_*t*_ and *A*_un_ denote the amount of total drug and analyzed amount of unentrapped drug in the nanosuspension, respectively.

### 2.7. Fourier Transform-Infrared (FT-IR)

Fourier transform-infrared (FT-IR) spectrums were obtained on a FT-IR spectrometer by the conventional KBr pellet method. The samples were grounded gently with anhydrous KBr and compressed to form pellet. The scanning and the resolution range were 400–4000 cm^−1^ and 4 cm^−1^, respectively.

### 2.8. Differential Scanning Calorimetry (DSC)

Thermograms of each sample were recorded by differential scanning calorimeter (TA Instruments Q 1000, Delaware, USA) equipped with intracooler and refrigerated cooling system. Each sample was placed in an aluminum pan and hermetically sealed with an aluminium lid. All measurements were performed at 5°C/min heating rate and nitrogen was purged at 50 mL/min through cooling unit.

### 2.9. Transmission Electron Microscope (TEM)

The morphology of the samples was observed using transmission electron microscope (TEM) with JEM-100S TEM (Philips Morgagni, 268). The samples were placed on a carbon coated copper grid to leave a thin film on the grid and negatively stained with 1% phosphotungstic acid. The sample grid was allowed to dry thoroughly at room temperature and was viewed with appropriate magnification.

### 2.10. *In Vitro* Drug Release Study

The drug release study was performed in pH 7.4 saline phosphate buffer (PBS) using the dialysis bag method. The dialysis membranes (thickness 0.025 mm, mol. wt. cut-off 12000–14000 Da) were soaked overnight in the dissolution media before study to ensure thorough wetting of the membrane. Both types of nanoparticle containing BBR (3 mg equivalent) along with 2 mL of the PBS were placed into the dialysis bag; two ends were tied and fixed by clamps. The dialysis bag was submerged into a beaker containing 75 mL of medium kept at 37 ± 0.5°C and stirred magnetically at 100 rpm. Samples were withdrawn at preset intervals with immediate replacement of equal volumes of the fresh medium to maintain sink condition. The samples were filtered through 0.22 *μ*m syringe filters and the BBR content was determined by HPLC method. A similar study was also conducted with pure BBR. All the studies were carried out in triplicate (*n* = 3).

### 2.11. Stability Study

Surface modified lyophilized nanoformulation was stored for 180 days at cool condition (8 ± 2°C) and characterized for PS, PDI, ZP, EE, and* in vitro* drug release studies during storage period.

### 2.12. Haemolysis Assay

Haemolysis experiments were performed according to the method of Bender et al. [41–43]. Human blood samples used were freshly obtained from authorised blood bank. Initially, 5 mL blood was centrifuged at 1600 rpm for 5 min. Supernatant plasma surface layer was removed and sediment RBC pellet was separated and washed thoroughly with normal saline solution. The RBC pellet was diluted with 25 mL of normal saline solution. Pure BBR and naked and surface modified NPs (equivalent to 10 *μ*g and 100 *μ*g of BBR) were added in 2 mL of RBC suspension separately. Similarly, naked and surface modified placebo NPs (i.e., equivalent weight to BBR loaded NPs) were also added to RBC suspension. Positive (100% lysis) and negative (0% lysis) control samples were prepared by adding equal volumes of Triton X-100 and normal saline, respectively, to RBC suspension. The samples were incubated at 37°C for 3 hrs. The samples were slightly shaken once for every 30 min to resuspend the RBCs and NPs. After 3 h, the samples were centrifuged at 1600 rpm for 5 min and 100 *μ*L of supernatants were incubated for 30 min at room temperature to allow haemoglobin oxidation. Oxyhemoglobin absorbance was measured spectrophotometrically at 540 nm. Haemolysis percentages of the RBC were calculated using the following formula:

(2) %Haemolysis =(abs  of  sample−abs  of  negative  control)(abs  of  positive  control−abs  of  negative  control).

### 2.13. LDH Assay

The lactate dehydrogenase (LDH) enzyme release from RBC was assessed spectrophotometrically using the LDH commercial kit (Span Diagnostics, India) [43]. All the samples were incubated in RBC suspension for 3 h as similarly described in haemolysis assay method. After that, the samples were centrifuged at 1600 rpm for 5 min and LDH released in supernatant was measured spectrophotometrically at 500 nm (U-1800, Hitachi). Equal volume of Triton X-100 was added to RBC suspension and treated as spontaneous control. The concentration of LDH released was calculated using the following formula:

(3) Lactate  dehydrogenase  (UL−1) =Abssample−AbscontrolAbsstandard×150,

where Abs_sample_ is the absorbance of the supernatant of the samples and Abs_control_ is the absorbance of the supernatant of the RBC suspension added to the substrate reaction. Abs_standard_ is the absorbance of supernatant of RBC suspension with LDH standard (150 UL^−1^, according to the manufacturer specifications). All samples were analyzed in triplicate (*n* = 3).

### 2.14. Platelet Aggregation

Platelet aggregation analysis was performed as described in the method of Bender et al. [43]. Peripheral blood smears were prepared after the incubation of whole blood with NPs to observe platelet changes due to the particle interaction. In brief, except positive and negative control, the samples likewise mentioned in haemolysis and LDH assay were incubated with whole blood for 3 hrs at 37°C. After incubation, peripheral blood smears were stained with Leishman's stain for 5-6 min (Span Diagnostics, India). Then, smears were rinsed by water and allowed to dry for few minutes. Dried smears were analyzed by optical microscope in immersion objective and images were captured using the digital system (Nikon Trinocular Microscopic Unit, Model E-200, Japan).

The quantitative evaluation of platelet aggregation was carried using haematological counter (ABX Micros 60, Horiba-ABX, India) for all the samples before and after incubation. All samples were analyzed in triplicate (*n* = 3).

## 3. Statistical Analysis

The results were expressed as mean ± SD. Statistical comparisons of the experimental results were performed by the Student's *t*-test and one-way ANOVA. In all cases, *P* values less than 0.05 were considered to be significant.

## 4. Results and Discussion

### 4.1. HPLC Analytical Method

Standard calibration curve of BBR was linear over the range 1–20 *μ*g/mL and the retention of BBR was 8 min. The regression equation was *y* = 23.044*x* − 0.9048 and mean correlation coefficient (*R* ^2^) was 0.9999. The accuracy (% of recovery) values of 2, 5, and 10 *μ*g/mL were 80.3%, 87.6%, and 88.4%, respectively. The coefficients of variation (CV) for intra- and interday precision were less than 10%, the LOQ was 15 ng/mL, and LOD was 5 ng/mL.

### 4.2. Preparation of Naked and Surface Modified Nanoparticles

Nanoparticles were prepared by nanoprecipitation method. The nanoparticles size range was around 200 nm. The nanoparticles represented homogeneous particle size, monopolydispersity index, and stable zeta potential (Table 1). The surface modification was done by 1% w/v concentration of vitamin E TPGS. The slight increase in PS and PDI and changing of zeta potential of NPs from negative to neutral region confirmed the successful surface modification (Table 1).

### 4.3. Particle Size, Polydispersity Index, and Zeta Potential

The pre- and postincubated naked and surface modified NPs in SBF pH 6.8 and 7.4 with or without sodium taurocholate (20 mM) and human plasma were characterized for their PS, PDI, and ZP (Tables 1 and 2). The PS, PDI, and ZP of naked and surface modified NPs were 190.71 ± 4.47 nm and 208.48 ± 1.07 nm; 0.116 and 0.166; −26.3 ± 0.8 mV and −10.32 ± 1.2 mV), respectively. After incubation of naked NPs in SBF pH 6.8 and 7.4 for 30 min, the particle size (2234.60 ± 458.63 and 3189.43 ± 87.12), PDI (0.509 and 0.721), and zeta potential (−02.35 ± 0.9 mV and −01.92 ± 0.7) increased, respectively. The increase in PS and PDI of naked NPs in pH 6.8 and 7.4 (mimics the microenvironments of intestinal region and systemic circulations) may be due to agglomeration of the naked NPs. Usually, the zeta potential of naked NPs (−26. mV) would be enough to prevent aggregation and maintain their stability at storage conditions. However, the changes in ZP of naked NPs from negative to neutral charge during incubation may have resulted in failure of its Brownian motion leading to aggregation and increase in particle size. This also reflects that the potential charge of particles in corresponding pH environment at which surface charge inspired electrostatic repulsion forces could not overcome the van der Waals attractive forces and eventually developed the tendency to agglomerate. Interestingly, insignificant changes in PS, PDI, and zeta potential were observed with naked NPs when incubated in SBF (pH 6.8) with STC of 20 mM. It may be due to the facilitation of appropriate alkaline environment by STC which prevents the agglomeration of negatively surface charged NPs. It has been reported that in alkaline environment negatively charged NPs are more stable than positively charged NPs [21, 44]. On other hand, significant difference in PS and PDI and insignificant changes in ZP were observed with naked NPs after incubation in human plasma. It may be due to the adsorption of protein corona on surface of NPs. A group of plasma proteins are called protein corona [45]. Further, the insignificant change in zeta potential indicated that the adsorbed protein corona was bonded to the surface of NPs due to hydrophobic nature and charge interactions but was not involved in agglomeration of NPs and subsequent instability. The vitamin E TPGS surface modified NPs showed insignificant differences in PS, PDI, and ZP and exhibited good stability (no agglomeration) at postincubation in similar conditions for the same time (30 min) due to its steric stabilization or stealth property. In surface modification, a tiny steric layer could be formed on surface of core particle which prevents the tendency of agglomeration due to electrostatic repulsion even at neutral zeta potential region.

### 4.4. Entrapment Efficiency

The EE of the BBR in naked NPs was found to be 87.12% and it was not significantly changed after its surface modification (Table 1). It is expected that the addition of surfactants (stabilizers) can increase the solubility of drugs in aqueous medium due to its surface tension reducing property. During the surface modification, it may cause leaching of entrapped drug from NPs during overnight incubation period which may lead to reduction in entrapment of drug. Typically, it has been reported that surfactant property of vitamin E TPGS could not enhance the BBR solubility in aqueous medium [46]. Thus, it may be the probable reason for insignificant difference in EE of BBR NPs after surface modification with vitamin E TPGS.

### 4.5. Fourier Transform-Infrared

**Figure 1:**
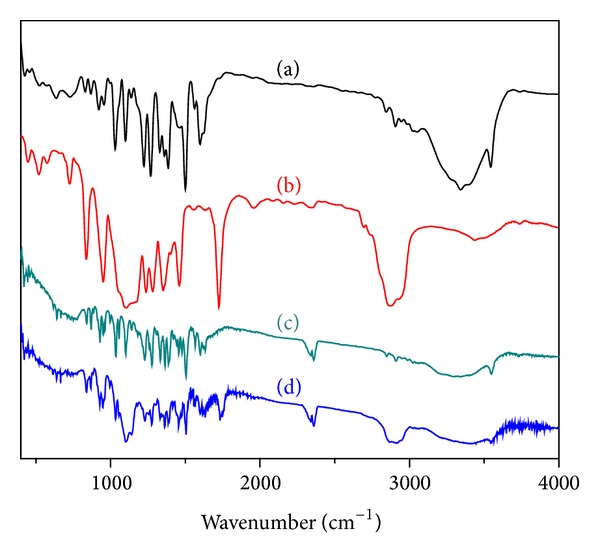
FT-IR spectra of (a) pure BBR, (b) PCL, (c) TPGS, and (d) surface modified NPs.

The FT-IR spectrum of pure BBR revealed the existence of a methoxyl group peak appearing at 2836 cm^−1^ and the iminium (C=N+) double bond peak at 1606 cm^−1^ (Figure 1(a)). Moreover, the peaks at 1569 cm^−1^ and 1506 cm^−1^ represent the aromatic C=C bending and furyl group, respectively. C–H peaks at 1719 cm^−1^ and 2857 cm^−1^ were identified in the IR spectra of PCL (Figure 1(b)) [47]. Characteristic carbonyl functional group peak at 1657 cm^−1^ along with other characteristic C–O–C stretching vibrations of the repeated –OCH_2_CH_2_ chain of TPGS was observed in the region of 1104–1268 cm^−1^ (Figure 1(c)) [48]. Absence of characteristic peak of BBR at 2836 cm^−1^ was confirmed successful entrapment of drug in nanoparticles (Figure 1(c)). Further, presence of broad peaks with slight shifting at 1740 cm^−1^, 2836 cm^−1^ of PCL and peaks at 1095–1227 cm^−1^ of TPGS due to hydrogen bonding indicated coexistence of PCL and TPGS in surface modified NPs. Thus, results demonstrated that surface modification was done without prominent chemical modification.

### 4.6. DSC

**Figure 2:**
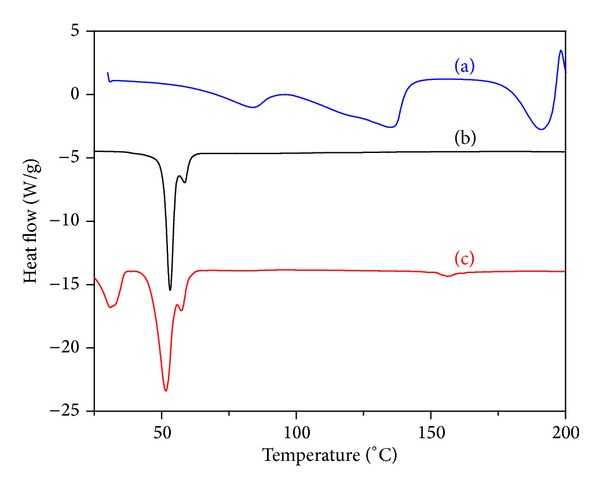
DSC thermograms: (a) pure BBR, (b) naked NPs, and (c) surface modified NPs.

DSC studies were performed to confirm the surface modification of NPs. It has been reported that the melting points of BBR, PCL, and vitamin E TPGS are around ~190°C, 55°C, and 40°C, respectively [49–52]. In this study, the melting point peak of BBR (~190°C) appeared in the thermogram of pure BBR along with other characteristic endothermic peaks (Figure 2(a)). The disappearance of the characteristic endothermic peak of BBR and appearance of PCL melting point peak at 54°C in thermograms of BBR loaded naked nanoparticles confirmed that the BBR existed in amorphous state (Figure 2(b)). In case of surface modified NPs, the appearance of additional sharp endothermic peak of vitamin E TPGS at 32°C without shift or formation of new peaks indicates the coexistence of TPGS in surface modified NPs (Figure 2(c)). Thus, it can be concluded that vitamin E TPGS was presented in surface modified NPs without chemical modification with drug and PCL. The results of FT-IR also supported the DSC results.

### 4.7. Transmission Electron Microscopy

**Figure 3:**
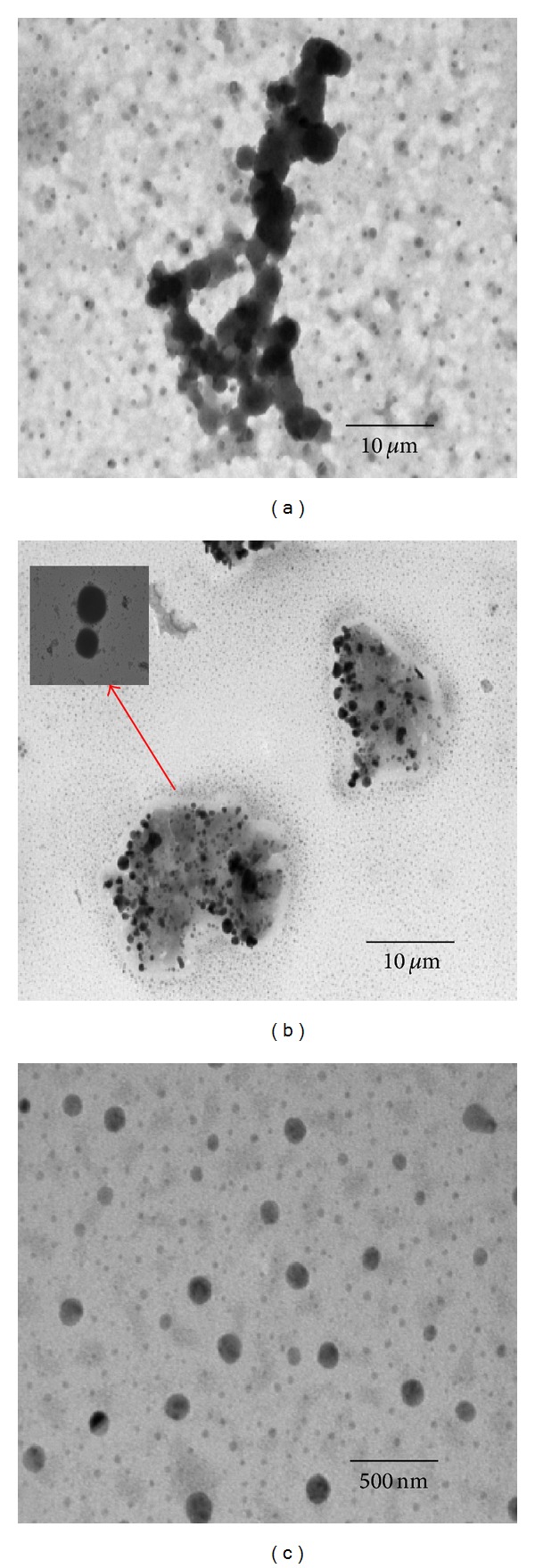
TEM images: (a) naked NPs in SBFs (pH 6.8), (b) naked NPs in plasma, and (c) surface modified NPs in pH 6.8 and 7.2 and in plasma.

Transmission electron microscopy was employed to verify the particle size results of both types of NPs which were observed at pre- and postincubation conditions. TEM images are shown in Figure 3. In TEM images, naked NPs had spherical shape and smooth surface whereas surface modified NPs exhibited steric layer on surface of the particle (Figure 3(c)). The appearance of steric layer surrounding the surface of particle not only characteristically confirmed the surface modification but also differentiated surface morphology between naked and surface modified NPs. Particle aggregates were observed in TEM image of naked NPs when they were incubated with SBF (pH 6.8) and corroborated with the results of particle size (Figure 3(a)). Slight rough surface and heterogeneous particles without aggregation were observed in TEM images of naked NPs which were incubated in plasma (Figure 3(b)). It may be due to addition of protein corona on surface of the naked NPs. However, TEM images correlated well with particles size results and showed addition of the protein corona. It might be appropriate reason for significant increase of the particle size of naked NPs. However, the agglomeration and heterogeneous particle sizes were not observed in TEM images of surface modified NPs at specified incubation conditions. The TEM images evidently demonstrated that the nonionic steric stabilization and surface hydrophilicity of vitamin E TPGS play a role in prevention of agglomeration and maintain stability at specified incubated conditions. The results of particle size and TEM images revealed that surface modification by vitamin E TPGS (1% w/v) prevented the agglomeration of BBR NPs when exposed to different SBF conditions.

### 4.8. *In Vitro* Drug Release Study

**Figure 4:**
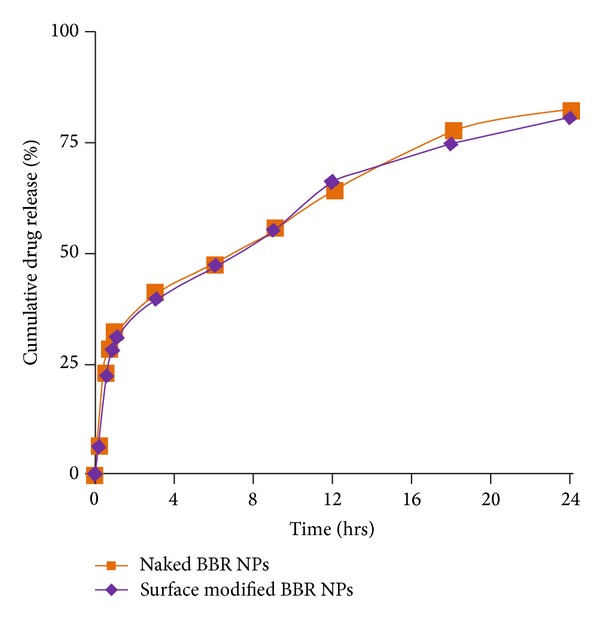
*In vitro* drug release profile of naked and surface modified BBR NPs.

*In vitro* release profiles of lyophilized naked and surface modified BBR NPs in saline phosphate buffer (pH 7.4) are shown in Figure 4. Both types of BBR NPs exhibited biphasic release pattern with an initial burst release of adsorbed BBR followed by sustained release of entrapped drug. In both types of NPs, nearly 82% of BBR was released in 24 h. The time required for half quantity of drug release (T50%) for both types of BBR NPs was found to be 6.34 h and 6.45 h, respectively. Insignificant difference in the drug release pattern of surface modified NPs compared to naked NPs was observed. Highly hydrophobic nature of PCL may retard the fast release of drug from the NPs. The release data is fitted into Korsmeyer and Peppas equation and the diffusion exponent (*n* value) was found to be 0.8791 for surface modified BBR NPs. The n value is the diffusion exponent which characterizes the transport mechanism and the value near to 0.89 indicates anomalous diffusion or non-Fickian diffusion [53, 54]. Hence, the release mechanism of surface modified BBR NPs was found to be non-Fickian diffusion.

### 4.9. Stability Study

Stability studies of lyophilized surface modified nanoformulation were carried out to evaluate the changes in parameters such as PS, PDI, zeta potential, EE, and drug release over a period of 180 days at cool temperature. There was no significant (*P* > 0.05) difference observed throughout the stability period in the above parameters (Table 3). Further, the* in vitro* drug release also did not exhibit significant difference in their drug release pattern during stability period.

### 4.10. Haemolysis Assay

Haemolysis is one of the rapid red blood cell burst processes. It may spontaneously occur at intolerable microenvironment condition. It is one of the significant parameters reflecting the incongruity with foreign material as well as acute toxicity. Occurrence of haemolysis mainly depends on the following impact such as net surface charge of the particle and chemical composition of the used material/excipient. It may also greatly affect the surface to volume ratio (macro, micro, and nano) as compared to their respective bulk materials [55, 56]. Thus, it becomes obligatory to evaluate the haemocompatibility of developed novel formulations. Particularly, it needs more attention in case of development of nanoparticulate delivery systems for drugs as nanoscale size of particle can interact easily with micron sized red blood cell and may initiate rapid haemolysis if materials are incompatible.

**Figure 5:**
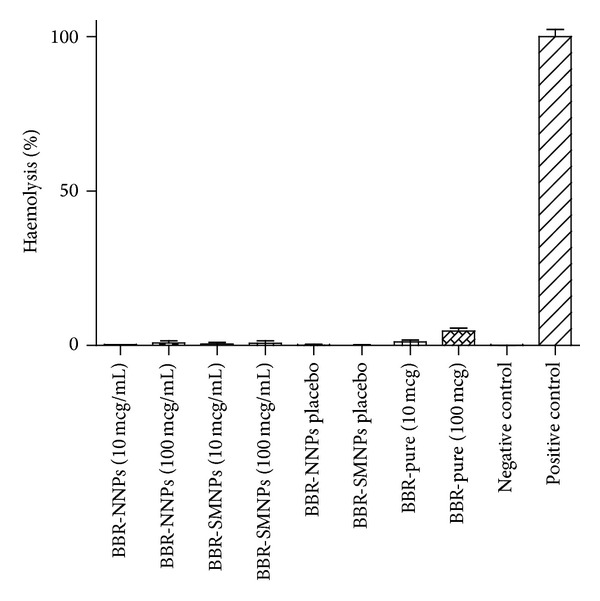
Haemolysis results of naked, surface modified drug loaded nanoparticles and placebos after incubation in SBFs. ∗BBR-NNPs: berberine loaded naked nanoparticles. BBR-SMNPs: berberine loaded surface modified nanoparticles.

The results of haemolysis assay are shown in Figure 5. Haemolysis test indicates that all samples had permissible haemolysis whereas pure BBR (100 *μ*g/mL) showed higher haemolysis but within the limits (<10%) when compared to positive control (assumed 100%) [41, 57]. The surface charge based interaction between positively charged drug and negatively charged red blood cells may be the appropriate reason for observed higher haemolysis in case of pure BBR at 100 *μ*g/mL. Both types of BBR loaded NPs equivalent to 100 *μ*g/mL did not show haemolysis because entrapped drug had facilitated lower exposure of drug to RBC even at similar bulk volume ratio of pure BBR. Previous reports indicate that interaction of cationic charged particles or drug with anionic charge RBC causes haemolysis. Electrorepulsion between negative charge of the naked NPs or neutral charge of the TPGS surface modified NPs with negatively charged blood cells may be a probable reason in avoiding the interaction between them and thus the subsequent haemolysis. As a result, surface modification with nonionic (neutral charge) nature excipients like TPGS may be beneficial in prevention of haemolysis and improves the NPs blood compatibility due to its noninterference in charge based interactions. The results of drug, naked, surface modified BBR and placebo NPs showed that net charge and exposure of the bulk volume of the NPs into RBC medium are found to be critical factors in haemolysis. The overall study results revealed that composition and concentration of both types of NPs with and without drug were found to be suitable for systemic administration.

### 4.11. LDH Assay

**Figure 6:**
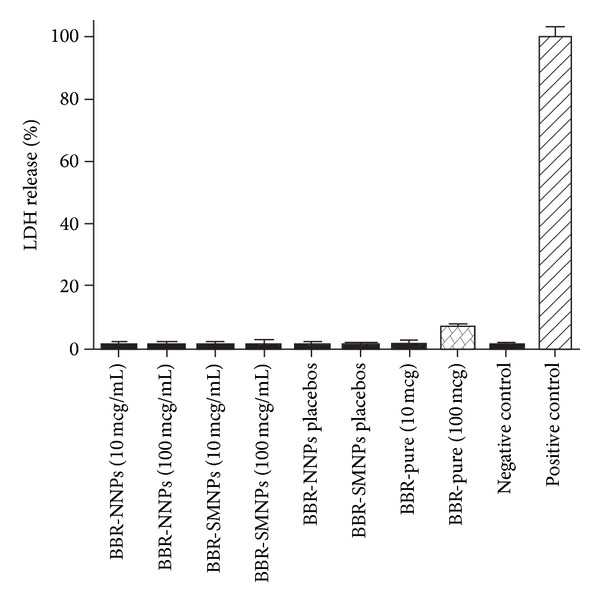
LDH assay results of naked, surface modified drug loaded nanoparticles and placebos after incubation in SBFs.

Erythrocyte membrane integrity was evaluated by LDH assay. This assay was intended particularly for evaluation of the influence of vitamin E TPGS on erythrocyte membrane integrity. The assay results are shown in Figure 6. All the samples exhibited insignificant increase in release of LDH enzyme compared to negative control. However, BBR (100 *μ*g/mL) sample released higher level of LDH enzyme when compared to other samples. It may be due to the bulk exposure of the pure BBR (100 *μ*g/mL) at specified RBC suspension volume and net charge interaction between them. This indicates that presence of higher quantities of BBR in blood may lead to adverse effects. On other hand, significant difference in LDH release between pure BBR and drug loaded NPs at 100 *μ*g/mL quantities revealed that encapsulation of drug in NPs and its surface modification not only improved the stability in relevant media but also reduced adverse effects associated with bulk exposure of drug in blood. Further, the obtained results supported the haemolysis results.

### 4.12. Platelet Aggregation

**Figure 7:**
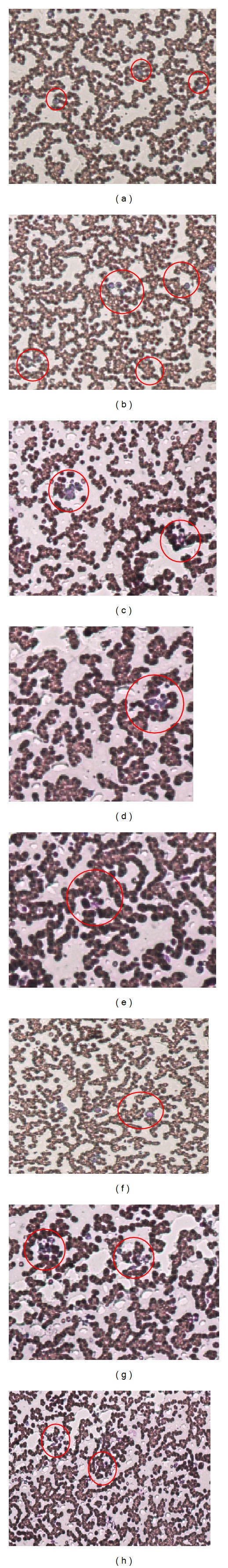
Platelet aggregation microscopic images of drug loaded naked, surface modified and placebos NPs (45x). (a) BBR-NNPs (10 mcg/mL), (b) BBR-SMNPs (10 mcg/mL), (c) BBR-SMNPs (100 mcg/mL), (d) BBR-NNPs (100 mcg/mL), (e) BBR-pure (10 mcg), (f) BBR-pure (100 mcg), (g) BBR-NNPs placebos, and (h) BBR-SMNPs placebos.

**Figure 8:**
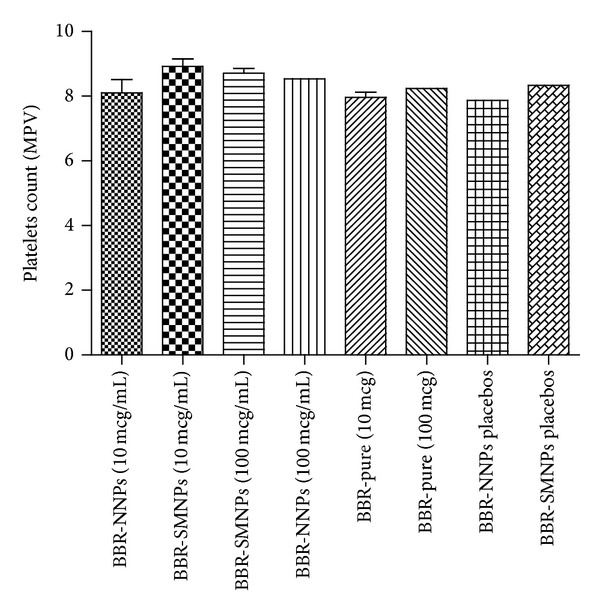
Platelet count results of naked, surface modified drug loaded and placebos NPs.

The interaction of platelets in blood samples with different NPs and pure BBR samples incubated was qualitatively studied by light microscopy. Normal saline treated blood samples were treated as a platelet aggregation control. Primarily, blood smears were prepared for the samples and stained as described earlier in Section 2.10. The stained samples were observed microscopically for qualitative visual confirmation. The microscopic images are shown in Figure 7. The platelets observed in microscopic images were indicated in round circles. There was no platelet aggregation in microscopic images of all samples. In certain conditions, exposure of higher quantity of drug may cause platelet aggregation. In this study even pure BBR (100 *μ*g) did not show such kind of platelet aggregation. It may be due to the antiplatelet aggregation property of BBR. Thus, the results confirmed that there was no impact of drug, polymer, and vitamin E TPGS on used quantities for platelet aggregation. In addition, the blood samples were quantitatively evaluated for platelet count by autohematological counter. The results are shown in Figure 8. The obtained platelet counts of all the samples did not show significant difference when compared to control.

Thus, the quantitative and qualitative results of above haemocompatibility studies reveal that excipients used, drug, and both types of NPs are hemocompatible and could be used for systemic administration.

## 5. Conclusion

BBR NPs were efficiently surface-modified using vitamin E TPGS (1% w/v). The quantitative and morphological characterization tools illustrated that surface modified BBR NPs were stable at different SBF and plasma compared to naked NPs. It is also demonstrated that STC (bile salt) played crucial role in prevention of particles aggregation at SBF (pH 6.8). It is suggested that administration of NPs via oral route may be preferable after meal whereas secretion of bile salts due to meals can help in absorption of NPs through intestine without aggregation. However, mechanistic evaluation is needed for further confirmation. The haemolysis, LDH assay, and platelet aggregation results confirmed that both types of NPs were compatible for systemic use. In addition, haemocompatibility study indicated that surface modifier, vitamin E TPGS, and its concentration (1% w/v) were found to be safe for systemic use. Overall, the study indicates that vitamin E TPGS surface modification is helpful in maintenance of stability of BBR nanoparticles at systemic and storage conditions together with long retention property and may have potential in drug delivery.

## Figures and Tables

**Table 1:** Results of naked and surface modified nanoparticles.

Type of nanoparticles	Particle size (nm)	PDI	Zeta potential (mV)	Entrapment efficiency (%)
Naked	190.71 ± 4.47	0.116	−26.3 ± 0.8	87.12
Surface modified	208.48 ± 1.07	0.166	−10.32 ± 1.2	86.98

**Table 2:** Results of naked and surface modified nanoparticles after incubation in SBFs at different pH's.

Incubation medium	Type of nanoparticle	Particle size (nm)	Zeta potential (mV)
Without incubation	Naked	190.71 ± 4.47	−26.3 ± 0.8
	Surface modified	208.48 ± 1.07	−10.32 ± 1.2

SBF pH 6.8	Naked	2234.60 ± 458.63	−02.35 ± 0.9
	Surface modified	206.23 ± 08.11	−09.32 ± 1.2

SBF pH 7.4	Naked	3189.43 ± 87.12	−01.92 ± 0.7
	Surface modified	212.84 ± 09.31	−09.17 ± 0.8

SBF pH 6.8 with bile salts	Naked	201.47 ± 21.10	−23.81 ± 1.4
	Surface modified	213.56 ± 02.36	−10.64 ± 3.1

Plasma	Naked	586.12 ± 51.23	−24.61 ± 2.2
	Surface modified	207.43 ± 06.17	−08.19 ± 0.7

**Table 3:** Stability studies of surface modified BBR NPs.

Parameters	Zero (0) month	Six (6) months
Particle size	208.48 ± 1.07	209.23 ± 1.21
Polydispersity index	0.166	0.164
Zeta potential	−10.32 ± 1.2	−10.37 ± 0.92
Entrapment efficiency (%)	86.98	85.71
